# The Significance of Histopathological Findings on Clinical Outcomes in Endoscopic Papillectomy with Endocut

**DOI:** 10.3390/jcm12216853

**Published:** 2023-10-30

**Authors:** Sayaka Miyamoto, Masahiro Serikawa, Yasutaka Ishii, Yumiko Tatsukawa, Shinya Nakamura, Juri Ikemoto, Yosuke Tamura, Kazuki Nakamura, Masaru Furukawa, Yumiko Yamashita, Noriaki Iijima, Koji Arihiro, Shiro Oka

**Affiliations:** 1 Department of Gastroenterology, Graduate School of Biomedical and Health Sciences, Hiroshima University, Hiroshima 734-8551, Japan; msayaka3@hiroshima-u.ac.jp (S.M.);; 2 Department of Pathology, Hiroshima University, Hiroshima 734-8551, Japan

**Keywords:** endoscopic papillectomy, ampullary adenoma, Endocut mode, specimen resection margin, residual tumor

## Abstract

This study aimed to evaluate primary clinical outcomes in patients who underwent endoscopic papillectomy (EP) using the Endocut mode while examining the pathological characteristics of the margin of the resected specimen. To this end, 70 patients who underwent Endocut EP were included. Resection margins were classified according to pathological findings as “negative”, “positive”, or “uncertain (difficult pathological evaluation)”. The effect of pathological resection margins on residual tumor recurrence rates was evaluated. The median follow-up was 47 months (range, 22–84). Eleven patients (15.7%) were diagnosed with residual tumors, ten of whom were diagnosed within 6 months after EP. The resection margins were pathologically negative in 27 patients, positive in 15, and uncertain in 28; residual tumors occurred in 5 patients (33.3%) in the positive group, 5 (17.9%) in the uncertain group, and 1 (3.7%) in the negative group. The patient in the negative group had familial adenomatous polyposis (FAP). Female sex, FAP, and uncertain or positive resection margins were significantly more common in residual patients (*p* = 0.009, 0.044, and 0.041, respectively). Pathological resection margins can be used to infer the residual tumor incidence, leading to early post-treatment of residual tumors.

## 1. Introduction

Endoscopic papillectomy (EP) is an endoscopic therapeutic technique for duodenal papillary tumors [1,2]. Since EP is less invasive compared to pancreatoduodenectomy (PD), a surgical treatment, it is widely performed as the first-line treatment for ampullary adenoma, mainly in high-volume centers [3,4,5]. Notably, technical standardization is almost established for EP procedures. A standard polypectomy snare is commonly used to firmly grasp the tumor and apply an electric current until the lesion is completely excised [6,7]. Owing to the advantage of reduced hemorrhagic complications, numerous institutions perform resection using electrosurgical units operating in Endocut mode [8,9], making the resection relatively safe.

A challenge associated with EP is its high residual rate after treatment. The post-EP recurrence rate has been reported to be 5–31% [2,10,11,12,13,14,15,16,17]. Early diagnosis of residual tumors after EP is extremely critical in clinical practice [18]. In cases of a residual adenoma, local treatments such as argon plasma coagulation (APC) cauterization or re-EP using a snare may be selected [19,20]. In cases of an adenocarcinoma lesion, surgery such as PD is selected [21,22]. However, when excision is performed in the mixed-wave/Endocut mode, tissue artifacts due to thermal coagulation are often observed, making it challenging to evaluate the presence or absence of remnants. Previous studies on EP have not addressed tissue degeneration and influence of thermal coagulation on the assessment of residual tumors in depth.

Therefore, this study aims to assess the primary clinical outcomes of all patients who underwent EP using the Endocut mode at our institution. Furthermore, we intend to investigate the impact of histopathological characteristics of resected specimen margins on the overall outcomes.

## 2. Materials and Methods

### 2.1. Patients

This study included 70 consecutive patients who underwent EP between January 2006 and June 2022 at Hiroshima University Hospital. After EP, all patients underwent endoscopic follow-up for over 1 year.

This study was approved by the Institutional Review Board of the Hiroshima University Hospital (No. E2021-2612) and was performed according to the guidelines of the Declaration of Helsinki.

### 2.2. EP Procedure

At our hospital, EP was considered suitable for patients diagnosed with adenomas via preoperative endoscopic biopsy and when endoscopic ultrasound (EUS) indicated that the tumor was confined to the papilla and did not extend along the sphincter of Oddi, bile duct, or pancreatic duct epithelium preoperatively.

Before performing EP, computed tomography was used to confirm the absence of metastasis, EUS was used to examine the vertical invasion and intraductal extension of the bile and pancreatic ducts, and endoscopic retrograde cholangiopancreatography was used to assess the morphology of the pancreatic ducts.

**Figure 1:**
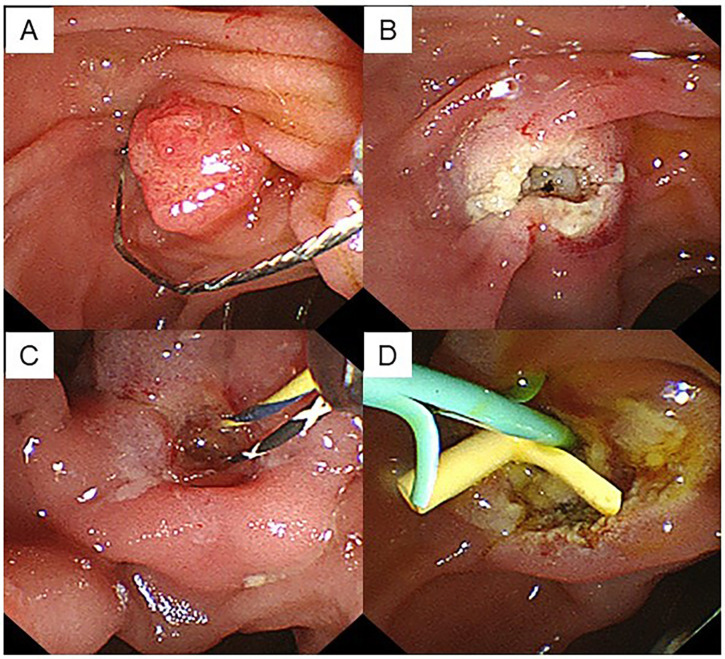
A case of ampullary adenoma that was indicated for endoscopic papillectomy. (**A**) EP was performed using a snare with a diameter of 13 mm. (**B**) No residual tumor was observed endoscopically after papillectomy. (**C**) Two guidewires were placed after cannulation of the bile and pancreatic ducts. (**D**) EP was completed by inserting a stent into both the biliary and pancreatic ducts.

EP resection was performed using a hard snare with a diameter of 13 mm (Captivator; Boston Scientific Corp., Marlborough, MA, USA) and a high-frequency electrosurgical generator ICC200 (Erbe, Tubingen, Germany) in Endocut mode until December 2020 and Vio3 in Endocut mode (Erbe, Tubingen, Germany) from January 2021, which comprised a pure cut current set at an output limit of 120 W and a soft coagulation current set at an output limit of 30 W (Figure 1A,B). En bloc resection of the ampullary adenoma was first attempted, and piecemeal resection was performed when en bloc resection was impossible. The EP was completed after confirming the absence of macroscopic residual lesions.

The specimen was collected immediately after resection, and endoscopic pancreatic stenting was performed using a 5-Fr stent to prevent pancreatitis (Figure 1C,D). When there were no serious adverse events, fluid intake was initiated from day 2, and oral intake was initiated from day 4 after EP. The endoscopic observation was conducted 7 days after EP, and the pancreatic and biliary stents were removed. The EP examinations were performed by three experienced endoscopists skilled in basic techniques related to endoscopic retrograde cholangiopancreatography (ERCP) and endoscopic resection.

### 2.3. Histopathological Evaluation

**Figure 2:**
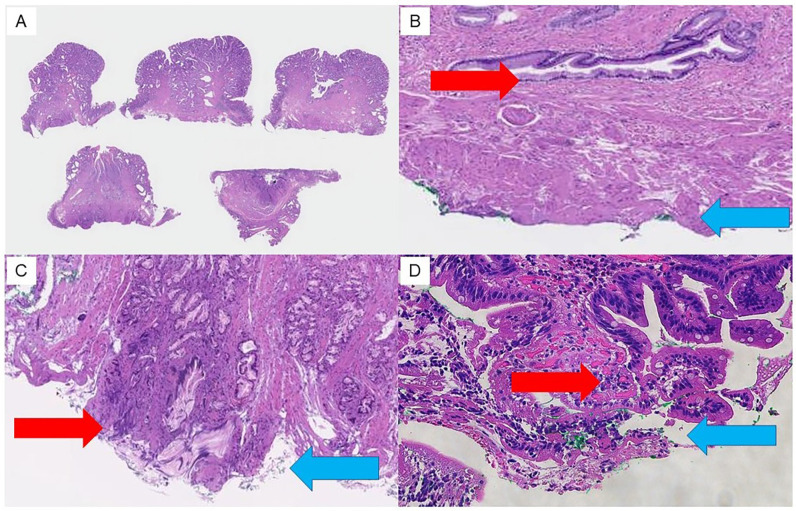
Findings of pathological margins in resected specimens. Resection margins are assessed pathologically as follows. (**A**) Resected specimens are fixed in formalin and sliced into 2 to 3 mm thick sections perpendicular to the resection surface. (**B**) “Negative” indicates no exposed tumor at the resection margin (VM/HM). (**C**) “Positive” indicates exposed tumor at the resection margin (VM/HM). (**D**) “Uncertain” indicates that the exposure of tumor cannot be assessed due to thermal degeneration. Red arrows indicate the vertical extension distance of the tumor. Blue arrows indicate the vertical resection margin.

The resected specimens were fixed in formalin and sliced into 2–3 mm thick sections perpendicular to the resection surface (Figure 2A), and vertical margin (VM) and horizontal margin (HM) were assessed as follows [22]: the margins were categorized into three categories, including “negative”, “positive”, and “uncertain” groups, depending on the pathological evaluation, allowing for an analysis of the frequency of each pathological finding of the resection margins and a comparison of the residual recurrence rates associated with the pathological resection margins. “Negative” indicates the absence of exposed tumor (Figure 2B), “positive” indicates presence of exposed tumor (Figure 2C), and “uncertain” indicates presence of tumor in the vicinity of the resection margin that was challenging to evaluate owing to tissue artifact caused by thermal coagulation at the VM, HM, or both surgical margins (Figure 2D).

Subsequently, to examine the factors related to thermal coagulation, we divided the patients into two groups, evaluable margin with negative/positive and unevaluable margin with uncertain, and examined the relationship with each clinicopathological factor.

### 2.4. Follow-Up Method after Endoscopic Papillectomy

**Figure 3:**
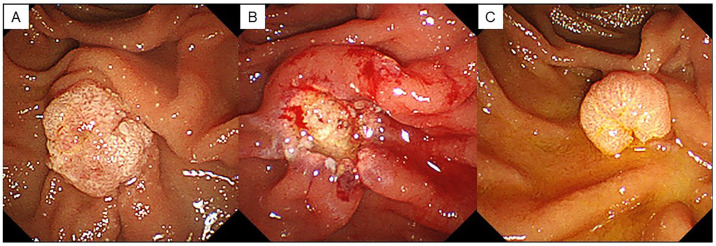
A case of residual lesion after EP. (**A**) Duodenoscopy showed an ampullary tumor. (**B**) An ulcer occurs after resection. No residual lesion is found endoscopically. (**C**) Endoscopic findings 3 months after EP show a residual lesion.

Endoscopic observations were performed at 3, 6, and 12 months after EP and then annually. Histopathological diagnosis was performed using an endoscopic biopsy when papillary redness, swelling, and granular mucosal changes were observed during endoscopic observation, and a residual tumor was suspected (Figure 3). When there were no findings suggestive of a residual lesion, a biopsy was not performed, and endoscopic observation was completed. Residual tumors were defined only in cases where the pathological findings on biopsy were adenoma or adenocarcinoma.

### 2.5. Statistical Analysis

All statistical analyses were conducted using JMP software (version 17; SAS Institute, Cary, NC, USA). Continuous variables are presented as medians (ranges) depending on the data distribution. The χ^2^ or Fisher’s exact test, when applicable, was used to compare frequencies. All statistical tests were two-tailed, and statistical significance was set at *p* < 0.05.

## 3. Results

### 3.1. Patient and Tumor Characteristics

We retrospectively collected the medical records and follow-up data of 70 patients. Table 1 presents clinical characteristics of the patients included in this study.

There were 55 males and 15 females, with a median age of 66.5 (range, 58–76.3) years, with 10 (13.9%) cases of familial adenomatous polyposis (FAP). Median (range) tumor diameter was 12 (range, 8.8–16.3) mm; en bloc resection was performed in 61 patients (87.1%) and piecemeal resection in 9. Early complications occurred in 15 patients (21.4%), including 2 hemorrhage, 4 pancreatitis, 8 cholangitis, and 1 stent migration cases. Late complications were observed in eight patients (11.4%). All complications were improved with internal medical treatment, and no EP-related deaths were recorded. The median (range) duration of the observation period after EP was 47.0 (22–84) months.

All patients underwent endoscopic biopsy for adenoma prior to the EP procedure, and the final pathological diagnoses after EP were as follows: adenoma, 56 cases (80%); high-grade dysplasia, 9 (12.9%); adenocarcinoma, 4 (5.7%); and adenomyomatosis of the duodenal papilla, 1 (1.4%). All adenocarcinomas were T1a cases. Endoscopic biopsy was accurate in 56 cases and underestimated in 13. The diagnostic concordance rate with the preoperative biopsy results was 80%.

### 3.2. Clinical Factors Associated with Pathological Findings of Resection Margin

Postoperative pathological margin evaluation of the resected specimen showed “negative” in 27 cases (38.6%), “positive” in 15 cases (21.4%), and “uncertain” in 28 cases (40.0%).

Among the positive cases, 10 were VM-positive, 4 HM-positive, and 1 both VM- and HM-positive. Among the uncertain cases, 3 cases were VM-uncertain, 3 HM-uncertain, and 22 cases both-uncertain.

Pathologically negative margins were confirmed in <40% of all cases resected using the Endocut mode at our institution. 

Between the two groups with negative/positive margins and unevaluable margin, no significant differences (*p* < 0.05) were observed in age, sex, FAP, resection method (en bloc resection or piecemeal resection), tumor size, or final pathological diagnosis (Table 2).

### 3.3. Residual Recurrence Rates Associated with Pathological Resection Margin

Table 3 presents clinical characteristics of patients with residual tumors. There were 11 cases (15.7%) of remnant after EP that were all diagnosed as adenoma using postoperative biopsy. The incidences of residual tumor per pathological diagnosis of resection margins were 3.7% (1/27) in the “negative” group, 33.3% (5/15) in the “positive” group, and 7.9% (5/28) in the “uncertain” group.

In the positive group, 30% (3/10) of VM-positive cases and 50% (2/4) of HM-positive cases had residual tumors. In the uncertain group, 22.7% (5/22) of both-margin-positive cases had residual tumors.

Patients with and without residual recurrence were compared according to age, sex, FAP, en block or piecemeal resection, tumor size, pathological final diagnosis, and resection margins. According to univariate analysis, female sex, FAP, and positive or uncertain resection margins were significant factors (Table 4).

### 3.4. Timing of Endoscopic Diagnosis of Residual Tumor and Post-Treatment after EP

Of the 11 patients with residual tumors, 8 patients (1 with negative margin, 3 with positive margins, and 4 with uncertain margins) were diagnosed during endoscopic follow-up after 3 months, and 2 patients (1 with positive margin, 1 with uncertain margin) were diagnosed during endoscopic follow-up after 6 months. Therefore, 10 out of 11 cases (90.9%) of residual cases were diagnosed as positive within 6 months after the endoscopic procedure. The median duration to the diagnosis of the residual tumor was 3 months (range, 3–50 months), and no association existed between the pathological findings of the resected margin and the time of recurrence (Table 3). One case diagnosed after 6 months was a case of FAP, which was diagnosed as a relapse 50 months after EP. The patient was diagnosed with a positive resection margin; however, during endoscopic follow-up 3 months later, no evidence of remnants was observed. The patient presented at 50 months after the procedure and was diagnosed with tumor recurrence via endoscopic biopsy; except for this case, no other patient experienced recurrence beyond 6 months after the procedure.

Six patients were treated endoscopically in five further resection sessions, and one underwent APC ablation. No residual or recurrent disease was observed after local treatment except in one case. One patient with adenocarcinoma and one with FAP were referred for surgery at the patients’ request, and three patients were followed up.

## 4. Discussion

In clinical practice, the Endocut mode is preferred in many institutions. For EP procedures, both Endocut and Autocut modes are currently available as electrosurgical generators [23,24]. As a feature of the incision wave, the Endocut mode automatically repeats the coagulation and incision waves alternately to proceed with the incision, whereas the Autocut mode performs a pure incision with the high-frequency waveform as a continuous wave [9]. There are no guidelines for recommending electrosurgical cutting modes in EP resection [2,25], and the selection of the cutting mode currently depends on endoscopist preferences. A systematic review comparing the incidence of early adverse events, including hemorrhage and pancreatitis, reported no difference between the two modes [26]; however, the results may be controversial because each report had different EP indications, incision settings, and definitions of hemorrhaging. In clinical practice, the Endocut mode is preferred in many institutions. Since our clinical experience showed lower immediate hemorrhage in the Endocut mode than in the Autocut mode, we believe that the Endocut mode is advantageous for EP.

Conversely, it has been pointed out that the Endocut mode may have a high incidence of postoperative pancreatitis [27,28]. This finding suggests that using coagulation waves in the Endocut mode promotes histological thermal degeneration. Tissue artifacts caused by thermal coagulation can obscure tumor margins and make it challenging to determine the depth of tumor vertical progression [20].

Previous studies on EP have not examined the degree of tissue degeneration in detail due to these thermal effects and the factors involved in degeneration. Therefore, the extent to which the Endocut mode degenerates the tissue and complicates the pathological evaluation of the resection margins is unknown. In our study, the “uncertain” case rate, which is difficult to evaluate pathologically, was as high as 40%. Notably, using the Endocut mode resulted in tissue artifacts in many cases. This appearance of artifacts is expected to correlate with tumor-related factors, such as tumor size and tumor extension, and technical factors, including the time required for electrosurgical incision; however, these data have not been demonstrated in other studies. In the present study, we compared several factors predicted to be related to patients with evaluable margins and those with unevaluable margins due to artifacts; however, no factors showed significant differences between the two groups. These factors should be prospectively investigated in future studies.

There is an obvious difference between residual and recurrent disease. Residual disease refers to the remaining lesion, whereas recurrence refers to new lesions that develop after resection [29]. In our study, the incidence of remnants after EP was 15.7%, consistent with the recurrence rate of 5–31% in previous studies [10,11,12,13,14,15,16,17]. However, previous studies did not clearly distinguish between residual and recurrent lesions. Distinguishing between these cases is often challenging. The diagnosis of residual tissue can be delayed for various reasons, including small residual tissue size and tissue burns from the procedure [30]. These two categories are now clinically differentiated according to the timing of lesion detection. Residual lesions are proposed to be defined as lesions found on surveillance endoscopy performed 3–6 months after EP, and recurrent lesions as lesions found ≥6 months after EP [31]. In our study, 10 of the 11 cases of residual recurrence were diagnosed within 6 months. The time of diagnosis defines these cases as residual tumors. One patient had a tumor diagnosed 50 months after EP, defined as recurrent disease.

In our study, there were no cases of recurrence except for one case of FAP. Kawashima et al. reported that the cumulative recurrence rates based on the Kaplan–Meier analysis after confirmation of remission were 3.9, 13.6, 16.9, and 22.5% at 1, 3, 5, and 10 years, respectively [32]. The reason for these differences was thought to be that we only indicated EP for cases that were confined to the papilla and excluded those with even a small amount of extension to the bile or pancreatic duct. These differences in the EP indication criteria were considered to be related to recurrence. Usually, residual tumors are supposed to present in the “positive” case in which the tumor is exposed to the specimen resection margins, but not all positive cases have remnants. In the cases in our study, only 33.3% of the pathologically “positive” margins had residual tumors. A possible reason for this discrepancy could be the mixed-wave thermal effect in the Endocut mode causing excisional necrosis of the residual tumors. Kim et al. reported heat denaturation during grasping with a snare during endoscopic resection of the early stage and reduced recurrence [33]. Similarly, in our study, 7.9% of the cases in the “uncertain” group were found to have residual disease. This incidence was slightly lower than that in the “positive” group; however, it was indicated that a certain rate of residual disease may also occur in the “uncertain” group. If the residual tumor can be predicted to some extent from the resected margins of the specimen, it will provide useful information during follow-up after EP.

In the “negative” group, the risk of occurrence of remnants is extremely low. We found one residual case, and the incidence of residual disease was 3.7% in our study. This “negative” case was a case of FAP, which was endoscopically diagnosed as a residual tumor 3 months after EP. As for FAP cases, a FAP case followed a different clinical course than that of other sporadic tumors. An endoscopic examination 3 months after the EP showed no remnants in this FAP case; however, recurrence was confirmed at a visit 50 months after the EP. These findings suggest that the behavior of papillary tumors with a FAP background differs from that of sporadic papillary tumors. Previous studies demonstrated that sporadic and FAP papillary tumors exhibit different recurrence patterns [29,34]. In these studies, patients with sporadic and FAP adenomas had similar recurrence risks during follow-up; however, those with FAP tended to have later recurrences than those with sporadic tumors. Therefore, even with negative resection margins, patients with FAP may require long-term follow-up because of the possibility of metachronous recurrence.

In conclusion, EP is an effective endoscopic treatment for duodenal papillary tumors, and it should be noted that postoperative remnants and recurrences may occur. Since pathological diagnosis suggests that additional treatment can be applied to residual or recurrent lesions after EP, efficient follow-up is required. This study showed that the pathological findings of the resection margins can be used to infer the residual incidence. Additionally, it revealed that endoscopic follow-up after EP requires different strategies for sporadic and FAP cases and that FAP may recur much later, even with negative resection margins, and long-term follow-up is required.

## Figures and Tables

**Table 1:** Baseline characteristics of 70 patients with ampullary tumors.

Characteristics	Values
Age (years)	67 (55–76)
Sex (male/female)	55/15
Familial adenomatous polyposis, *n* (%)	10 (13.9%)
Clinical presentation, *n* (%)	
Incidental findings	55 (78.5%)
Symptomatic	15 (21.4%)
Tumor size (mm)	12 (8.8–16.3)
Resection, *n* (%)	
En block	61 (87.1%)
Piecemeal	9 (12.9%)
Early complications, *n* (%)	15 (20.8%)
Pancreatitis/cholangitis/bleeding/stent migration	4/8/2/1
Late complications, *n* (%)	8 (11.1%)
Pancreatitis/biliary stone/cholangitis	4/3/1
Final pathological diagnosis, *n* (%)	
Adenoma	56 (80.0%)
High-grade dysplasia, adenocarcinoma	13 (18.6%)
Adenomyomatosis	1 (1.4%)
Resection margin, *n* (%)	
Negative	27 (38.6%)
Positive	15 (21.4%)
Uncertain	28 (40.0%)
Follow-up period (months)	47 (22–84)
Data are expressed as number (percentage) or median (interquartile ranges)	

**Table 2:** Factors associated with pathological assessment of resection margin.

	Evaluable Margin (*n* = 42)	Unevaluable Margin (*n* = 28)	*p* Value
Age (years)	68 (60–77)	63 (53–74)	0.332
Sex (male/female)	34/8	21/7	0.567
Familial adenomatous polyposis, *n* (%)	4 (9.5%)	6 (21.4%)	0.183
Resection, *n* (%)			0.468
En block	38 (90.5%)	23 (82.1%)	
Piecemeal	4 (9.5%)	5 (17.9%)	
Tumor size (mm)	12 (9–15)	10.5 (8–17.5)	0.276
Pathological diagnosis, *n* (%)			0.854
Adenoma	34 (81.0%)	22 (78.6%)	
High-grade dysplasia, adenocarcinoma	7 (16.7%)	6 (21.4%)	
Adenomyomatosis	1 (2.4%)	0	

**Table 3:** Clinical characteristics of patients with residual tumor.

Case	Age (Years)	Sex	FAP	Size (mm)	Pathological Final Diagnosis	Resection Margin	HM/VM	En Block/Piecemeal Resection	Pathological Residual Diagnosis	Post-Treatment	Time to Recurrence (Months)
1	53	M	+	5	Adenoma	Negative	-/-	En block	Adenoma	PD	3
2	76	M	-	15	Adenoma	Positive	-/+	En block	Adenoma	EP	3
3	73	M	-	15	Adenoma	Positive	-/+	Piecemeal	Adenoma	EP	3
4	37	F	+	18	Adenoma	Positive	-/+	En block	Adenoma	Follow-up	3
5	82	F	-	10	Adenoma	Positive	+/-	En block	Adenoma	EP	6
6	27	M	+	6	Adenoma	Positive	+/-	En block	Adenoma	EP	50
7	59	F	-	10	Adenoma	Uncertain	X/X	Piecemeal	Adenoma	APC	3
8	81	F	-	25	Adenoma	Uncertain	X/X	Piecemeal	Adenoma	Follow-up	6
9	66	F	-	10	Adenocarcinoma T1a	Uncertain	X/X	En block	Adenoma	PD	3
10	83	M	-	16	Adenocarcinoma T1a	Uncertain	X/X	En block	Adenoma	EP	3
11	88	M	+	12	Adenocarcinoma T1a	Uncertain	X/X	En block	Adenoma	Follow-up	3

APC, argon plasma coagulation; EP, endoscopic papillectomy; F, female; FAP, familial adenomatous polyposis; M, male; PD, pancreaticoduodenectomy.

**Table 4:** Comparison of clinical characteristics based on the residual recurrence.

	No Residual Recurrence (*n* = 59)	Residual Recurrence (*n* = 11)	*p* Value
Age (years)	66 (58–74)	73 (37–82)	0.744
Sex (male/female)	50/9	5/6	0.009
Familial adenomatous polyposis, *n* (%)	6 (10.2%)	4 (36.4%)	0.044
Resection, *n* (%)			0.143
En block	53 (89.8%)	8 (72.7%)	
Piecemeal	6 (10.2%)	3 (27.3%)	
Tumor size (mm)	12 (8–15)	10 (7–17.5)	0.931
Pathological diagnosis, *n* (%)			0.513
Adenoma	48 (81.4%)	8 (72.7%)	
High-grade dysplasia, adenocarcinoma	10 (16.9%)	3 (27.2%)	
Adenomyomatosis	1 (1.7%)	0	
Resection margin, *n* (%)			0.041
Negative	26 (44.1%)	1 (9.1%)	
Positive/Uncertain	33 (55.9%)	10 (90.9%)	

## Data Availability

The data that support the findings of this study are available on request from the corresponding author. The data are not publicly available due to their containing information that could compromise the privacy of research participants.
